# Coreceptor use in nonhuman primate models of HIV infection

**DOI:** 10.1186/1479-5876-9-S1-S7

**Published:** 2011-01-27

**Authors:** Silvana Tasca Sina, Wuze Ren, Cecilia Cheng-Mayer

**Affiliations:** 1 Aaron Diamond AIDS Research Center, 455 First Ave, 7th Floor, New York, New York, USA

## Abstract

SIV or SHIV infection of nonhuman primates (NHP) has been used to investigate the impact of coreceptor usage on the composition and dynamics of the CD4+ T cell compartment, mechanisms of disease induction and development of clinical syndrome. As the entire course of infection can be followed, with frequent access to tissue compartments, infection of rhesus macaques with CCR5-tropic SHIVs further allows for study of HIV-1 coreceptor switch after intravenous and mucosal inoculation, with longitudinal and systemic analysis to determine the timing, anatomical sites and cause for the change in envelope glycoprotein and coreceptor preference. Here, we review our current understanding of coreceptor use in NHPs and their impact on the pathobiological characteristics of the infection, and discuss recent advances in NHP studies to uncover the underlying selective pressures for the change in coreceptor preference in vivo.

## Introduction

Animal models have always been considered powerful tools for studying the modality of transmission and pathogenesis of human diseases, and for testing the efficacy of novel drugs and vaccines. They afford opportunities to closely monitor the natural course of the disease, with frequent sampling of blood and tissue compartments that are not easily amenable or accessible in humans. Besides their use in elucidating the pathogenic mechanisms of disease, appropriate animal challenge models play key roles in basic vaccine discovery, potentially providing valuable information on the immunogenicity and efficacy of vaccine concepts, and advancing candidate vaccines into human clinical trials.

The challenge in establishing animal models for HIV-1, however, is that the virus does not replicate in most animal species tested [1]. The exceptions are chimpanzee and gibbon apes [2,3], but experimental HIV-1 infection of chimpanzees is typically non-pathogenic, with only rare animals developing AIDS-like symptoms after prolonged incubation period [4-6]. Furthermore, these apes are endangered and costly to maintain, limiting their use for research purpose [7]. The AIDS pandemic originally arose as a consequence of zoonotic transmission of simian immunodeficiency viruses (SIVs) from African non-human primate (NHP) species to humans [8]. Phylogenetic analyses indicated that HIV-1 and HIV-2 emerged following transmissions of SIVcpz from chimpanzee and SIVsmm from sooty mangabeys (SM), respectively [9,10]. The similarities between SIV and HIV with respect to genomic structure and biological features renders infection of various macaque species with SIVs, or with chimeric viruses containing both SIV and HIV sequences (SHIVs), the most relevant animal models to study HIV-1 infection and AIDS.

## SIV infection of African and Asian monkeys and coreceptor usage

Over 40 distinct SIVs are found naturally in African NHPs [11]. Infection of natural hosts such as SMs, African green monkeys (AGMs) and mandrillis with SIVs is typically nonpathogenic despite sustained high levels of virus replication [12-15]. Development of AIDS has only been observed in one SM and in one mandrill, after 18-year-incubation, a period exceeding the normal lifespan of wild primates [16,17]. SIVcpz, the precursor for HIV-1, however, is pathogenic in free-ranging chimpanzees. Infected chimps in the wild were 10-16 times more likely to die in any year than those who remained uninfected [18], challenging the prevailing notion that all natural SIV infections are non-pathogenic. In contrast to most natural infections, accidental or experimental transmission of SIVs from SMs to Asian non-natural NHP hosts such as rhesus macaques resulted in progressive infection and AIDS-like symptoms, giving rise to SIVmac [19]. Indeed, Indian rhesus macaques (IndRMs; *Macaca mulatta*) infected with SIVmac and chimeras encoding the HIV-1 envelope or reverse transcriptase are the best characterized and most widely used animal models of AIDS [20]. Although the tempo of virus replication and disease progression in SIV infected IndRMs is significantly accelerated compared to human HIV infection, the pathobiology and clinical symptoms of SIV and HIV infection are remarkably similar [21]. Considerable efforts have been made to understand the similarities and differences between pathogenic and non-progressive SIV infections, with the hope of uncovering new host defenses that will guide conventional AIDS vaccine development. But with a decrease in availability of IndRM, alternative models of pathogenic infection, such as Chinese RMs, cynomolgus macaques (*Macaca fascicularis*), and pigtailed macaques (PTMs, *Macaca nemestrina*) are also being developed [20,22]. While SIVmac and SHIV infection in Chinese RMs and cynomolgus macaques appear to be more attenuated [23], PTMs are more susceptible, with cases of AIDS reported after experimental infection with SIVsmm, SIVagm, SIVlhoest from l’hoest monkeys, and SIVsun from sun-tailed monkeys [24-26]. The absence of the TRIM5α restriction factor that blocks infection by inactivating incoming capsids in PTMs explains the higher susceptibility of these NHPs to different SIVs [27-30].

Similar to HIV-1, most SIV strains use CCR5 as their primary coreceptor [31-33], despite a paucity of CD4+CCR5+ T cells in the natural hosts [34]. The notable exception is the red capped mangabey. Due to a high frequency of a 24 bp deletion in the CCR5 gene, SIVrcm uses CCR2, a chemokine that is expressed at low levels on CD4+ T cells but at higher levels on macrophages, for entry [35]. Nonetheless, the coreceptor usage profile of SIV is broader than that of HIV-1 in coreceptor-transfected cell lines [36,37] and PBMCs [38]. Among those reported to be used by SIV, some as efficiently as CCR5, are chemokine receptors that include CCR3, CCR4, CCR8, CXCR6/STRL33/Bonzo, various G-protein coupled receptors (ChemR23, GPR1, GPR15/Bob, RDC1, APJ), and the formyl peptide receptor FPRL1. Whether these alternative coreceptor pathways play a role in SIV infection and pathogenesis in vivo remains to be fully elucidated, but two recent reports provide some insights. In a study to examine the dynamics of in vivo replication of the CCR2- and macrophage-tropic SIVrcm in PTMs, expansion of coreceptor usage to CCR4 was observed, and this was associated with selective depletion of memory CD4+ T cells [39]. More recently, a novel 2 base pair deletion (Δ2) in the sooty mangabey CCR5 gene that resulted in a truncated CCR5 molecule has been identified. This mutant protein is not expressed on the cell surface and does not support SIV entry in vitro [40]. But SIV prevalence was only moderately lower in homozygous CCR5Δ2 mutant animals compared to heterozygous and wild type animals, and plasma viral loads were moderately reduced (0.48 log_10_) in CCR5Δ2 infected animals compared with wild type animals, demonstrating that CCR5-independent entry pathways are used by SIVsmm in naturally infected SMs.

In contrast to HIV-1, however, CXCR4 usage by SIVs in models of nonpathogenic and pathogenic infection is rarely observed. Only an isolate obtained from mandrills (SIVmnd; [41]) and one from AGM (SIVagm.sab; [42]) were found to use this coreceptor in addition to CCR5 for entry, and expansion to CXCR4-use was documented in two studies of SIVsm/SIVmac infection. In examination of six SIV seropositive SMs with AIDS-defining CD4+ T cell levels, variants with expanded coreceptor usage that included CXCR4 and CCR8 were identified in two mangabeys [43]. Emergence of the multitropic (R5/X4/R8) variants coincided with severe CD4 decline in the blood, lymph nodes and gut-associated lymphoid tissue (GALT), but the infected mangabeys remained clinically healthy for >8 years. Sequence comparison of the multitropic SIVsmm variants to viruses isolated prior to the change in coreceptor preference shows amino acid substitutions and insertions in the V3 loop that increase the overall net positive charge of this envelope region. But genetic studies to demonstrate that these changes in the V3 loop of SIVsmm are sufficient to confer expansion to CXCR4 and CCR8 use are lacking. Expansion to CXCR4 usage has also been reported in a SIVmac239 infected macaque [44,45]. Compared to the inoculating virus, the lymph node derived variant (SIVmac155T3) has a 10-fold reduction in CCR5 use that is accompanied by acquisition of CXCR4 utilization. Infection of RMs with SIVmac155T3 resulted in rapid and profound circulating and lymph node CD4+ T cell loss [46], similar to observations made in X4 SHIV infected rhesus monkeys (see below). SIVmac155T3 harbors 22 amino acid differences in the envelope glycoprotein compared to the inoculating virus, SIVmac239, including five substitutions and one insertion in the V3 loop [44], but the genetic determinant for expansion to CXCR4 use of this virus remains to be determined.

## Impact of coreceptor usage on SIV and SHIV infection of Asian macaques

Because the differences in SIV and HIV-1 protein structures might limit the utility of the SIV/macaque model to study the impact of genetic variations on disease outcome, SHIVs containing both SIV and HIV sequences have been engineered. The use of SHIVs that express the envelopes of diverse HIV-1 strains as inoculating viruses increases the relevance of NHP models, as the impact of HIV-1 envelope-determined properties such as coreceptor usage on viral transmission, persistence and pathogenesis can be assessed [47]. Furthermore, pathogenic envelope SHIVs could facilitate direct clinical analysis of HIV-1 Env-based candidate vaccines. Since most HIV-1 transmissions are initiated with R5 viruses, pathogenic and mucosally transmissible R5 SHIVs would be the preferred tools to assess and advance these vaccine concepts. For these reasons, R5, dual-tropic as well as X4 clade B [48-55], clade C [56-60], clade A and clade E SHIVs [61,62] have been constructed and assessed for cell and tissue tropism, viral persistence, anti-viral immune responses and disease progression in NHPs. These SHIV constructs do not readily induce disease in RMs. But through serial in vivo passages and adaptation, several X4 [63,64], dual-tropic [65,66], and R5 [54,67] subtype B envelope SHIVs that induce AIDS have been generated. R5 subtype C SHIVs that are highly replication-competent and mucosally-transmissible have also be obtained and are being tested for pathogenicity [68]. Interestingly, while dual-tropic SHIVs can use both CCR5 and CXCR4 efficiently on coreceptor-transfected cell lines, they use almost exclusively CXCR4 to enter and spread in cultured rhesus peripheral blood mononuclear cells and macrophages [38,69,70].

The most extensively studied SHIVs thus far use the CXCR4 coreceptor. X4 SHIVs can be transmitted intravenously (i.v.), as well as intrarectally (i.r.) or intravaginally (ivag) in RMs, demonstrating that there is no intrinsic barrier to mucosal transmission and amplification of X4 viruses. The clinical course of X4 SHIV infection in NHPs, however, differs dramatically from that of macaques infected with SIV and R5 SHIVs. Rapid and severe depletion of CXCR4+ target T cells in the peripheral blood, thymus and secondary lymphoid tissues was observed in X4 SHIV infected animals, with high sustained viremia and progression to disease in 12-30 weeks [63-65,71,72]. Similar to observations made in RMs infected with X4 SIVmac155T3 [46], CXCR4+ naive CD4+ T cells that are enriched in secondary lymph nodes were selectively depleted early in X4 SHIV infected monkeys, with elimination of central memory CD4+ T cells during post-acute infection [70,73]. Despite the severe and irreversible loss of CD4+ T cell populations, plasma viral load remained high in the inoculated rhesus monkeys, sustained by infected tissue macrophages [74]. However, while HIV-1 macrophage infection is generally associated with CCR5 use [75], acquisition of macrophage tropism by X4 SHIV in late-stage infected macaques is not accompanied by a change in coreceptor usage [76]. In a study that followed the evolution of HIV-1 envelope over time in PT macaques infected with X4 SHIVs [77], most isolates were also found to maintain CXCR4 use. The exception was an envelope protein obtained from the cerebral spinal fluid of an infected macaque that developed severe CD4+ T cell loss and AIDS. This brain-derived Env was found to use CXCR4 very inefficiently, but was able to use CCR2b, and to a lesser extent CCR3, STRL33 and APJ to infect cells. Furthermore, this envelope protein did not use CCR5, but mediate infection of macrophages. Thus, even though CXCR4 is very rarely used by SIV, it functions as an efficient receptor for SHIVs in macaques.

In contrast to X4 SHIV, R5 SHIV infected IndRMs experienced a variable but detectable plasma viremia, with some developing a rapid progressor clinical course. Furthermore, acute pathogenic R5 SHIV infection, similar to HIV or SIV infection [46,78-83], results in a more gradual and moderate loss of CD4+ T cells in peripheral blood and secondary lymphoid tissues, but dramatic depletion of CD4+ effector memory T cells that reside in extra-lymphoid immune effector sites such as the gut, lung and genital tract [54,67,68]. Thus, differential target cell selectivity of the two viruses [46,70], coupled with the difference in tissue distribution of the CD4+ target T cell subsets within the host [73,84,85], largely explains the distinct pattern and sites of CD4+ T cell depletion in RMs infected with X4 or R5 viruses. That this process is inextricably linked to coreceptor usage was further demonstrated in a study using isogenic SHIVs that differ only in the gp120 V3 loop sequences and in coreceptor preference [73]. Infection of RMs with pathogenic SIVs and SHIVs of different coreceptor usage, therefore, recapitulates key features of HIV infection and pathogenesis in humans: R5 SIV/SHIV infection induces a disease course that is more similar to that which occurs during most human infections with HIV-1, while X4 SIV/SHIV infection reproduces the precipitous peripheral CD4+ T cell decline observed in patients infected with X4 HIV-1 isolates or in late stage disease concomitant with the emergence of X4 virus. Coreceptor directed targeting results in dramatically different changes in the CD4 T cell subset composition of X4 and R5 SIV/SHIV infected animals, explaining the distinctive clinical courses induced by each virus.

## Coreceptor switch in R5 SHIV infected rapid progressor macaques

In ~50% of HIV-1 subtype-B infected individuals, the coreceptor usage of the virus changes from a preference for CCR5 to a preference for CXCR4 over the course of infection [86]. As noted above, expansion or switch to CXCR4 use is rarely observed in SIV infection of RMs, but several cases of tropism switch have been reported in R5 SHIV infection. Coreceptor switching was documented during infection of RMs inoculated with R5 SHIV_SF162P3N_ by the intravenous [87,88] and the intrarectal route [89]. The IV and mucosally infected macaques in which X4 virus evolved and emerged are rapid progressors (RPs) that experienced high set-point viral loads, undetectable or transient anti-viral antibody response, and progression to AIDS within 3-6 months post-infection. Viruses recovered from one of three RPs inoculated with the CCR5-tropic SHIVAd8 lineage viruses were also shown to acquire the capacity to use CXCR4 [54]. Although rare, rapid disease progression has also been reported in HIV-1 infected individuals [90-93], many of whom were found to harbor X4 viruses [94,95]. In contrast, while approximately 20% of SIVmac/SIVsm infected RMs become RPs, R5-to-X4 switching has not been documented in these rapid progressing macaques [96,97]. As coreceptor usage by SIV is broader than that for HIV-1, these findings lend further support to the notion that the use of other seven-transmembrane receptor(s) besides CCR5 and CXCR4 may indeed be relevant for SIV infection in vivo.

Consistent with findings in HIV-1 infected individuals that acquire X4 viruses [98-100], sequence changes in the V3 loop of envelope gp120 that increased the net charge of this region determine the phenotypic change from R5-to-X4 in R5 SHIV infected macaques [54,87-89]. These include insertions of basic amino acids upstream of the GPGR crown of the V3 loop, as well as replacement of the serine residue at position 11 of the V3 loop with positively charged amino acids, suggesting that evolution pathways to acquisition of CXCR4 use overlap in the two hosts. Similar envelope changes during disease progression in HIV-infected humans and SHIV-infected macaques have previously been reported, indicative of common selection pressures [101,102]. With regard to coreceptor switch, the observation that the envelope V3 loop sequences required for tropism switch in RP macaques are similar to those in infected humans who usually have developed neutralizing antibodies is noteworthy, since it argues that humoral immunity may not be a main selection pressure for change in coreceptor preference. Additionally, the V3 loop sequence mutations that resulted in tropism switch in R5 SHIV_SF162P3N_ infected RP macaques differed from those required to confer expanded CXCR4 use to the parental SF162 envelope in tissue culture systems [103-105], implying that the selective pressures for X4 virus evolution differed in vivo and in vitro. Because both systems lack antiviral antibody response, this selection factor also cannot explain the difference in sequence change required for adaption of the SF162 envelope to function with CXCR4 in vivo and in vitro. Besides humoral immunity, changes in target cell populations during the course of infection has also been proposed as playing a role in driving coreceptor switching [106,107]. Thus, it is conceivable that the need to escape this selection pressure, while maintaining viral fitness and efficient coreceptor usage on diverse tissue cells of the monkey host, accounts for the difference in sequence requirement for tropism switch in vivo and in tissue culture systems. However, X4 virus emergence in R5 SHIV infected RP macaque occurs at a time when peripheral CD4+ T cell count is >200 per microliter blood, suggesting that CD4+ target T cell limitation is also not a main selection pressure for change in coreceptor preference. A lack of correlation between the percentage of peripheral CCR5+CD4+ target T cells or CCR5 genotype with development of CXCR4-using viruses in HIV-1 infected individuals had also been reported [108,109]. Further studies in this animal model of coreceptor switch therefore may uncover novel selective pressures that lead to the evolution of CXCR4-using HIV variants in some infected individuals.

## Phenotypic characteristics of switch variants in R5 SHIV infected rhesus macaques

Similar to observations made in humans [110-112], the evolutionary process in infected macaques transitions through dual-tropic variants capable of using both coreceptors, albeit with reduced efficiency compared to the inoculating R5 virus and the final X4 variant [113]. The findings that SIVmac155T3 as well as the multitropic R5/X4/R8 SIVsmm variants still function with the CCR5 coreceptor while expanding their coreceptor utilization further illustrate the role of transitional intermediates in the pathway to coreceptor switch in NHPs. Furthermore, more than one R5-to-X4 evolutionary pathways were identified in some R5 SHIV infected RP macaques, giving rise to distinct X4 and dual-tropic variants which had a preference for CCR5 coreceptor (dual-R tropic) or a preference for CXCR4 coreceptor (dual-X tropic) [89,114]. Further characterization of these R5X4 intermediate viruses should provide a better understanding of the costs and benefits associated with switch in vivo, and properties of the transitional intermediates that allow them to eventually outgrow and amplify.

Akin to HIV-1 infection in humans [115], the emerging dual-tropic and X4 viruses are highly susceptible to antibody neutralization compared to the early or co-existing R5 viruses, in particular to soluble CD4 (sCD4) and anti-V3 loop antibodies. In this regard, varying frequencies of X4 viruses have been reported throughout the course of HIV-1 natural infection [116], but their dominance is seen only towards the end stage of disease. These findings, coupled with the observation that dual-tropic and X4 virus evolution is observed in R5 SHIV infected RP macaques with undetectable antiviral antibody responses, lend support to the notion that X4 emergence is the result, rather than the cause, of immune failure [117-119]. Thus, while antiviral antibody response may not be a main driving force for R5-to-X4 evolution, it is a major obstacle to X4 emergence and expansion. Interestingly, and consistent with reports for HIV-1 [120,121], V3 sequence changes that confer CXCR4 usage are also sufficient to determine increase sCD4 sensitivity of the virus [113], suggesting that the early steps in the R5-to-X4 evolution process in the RP macaques may require the same conformation changes that renders the virus neutralization sensitive. Higher sensitivity of viruses to neutralization with sCD4 and anti-V3 loop antibodies implies greater exposure of the CD4 and chemokine receptor binding sites that are usually sequestered away from the humoral immune response [122-124], indicative of a more “open” and less constrained envelope conformation. As envelope structural constraints have been suggested to limit the pathways available for coreceptor switching [110,125,126], it is tempting to speculate that an “open” and less constrained envelope conformation would be more accommodating for mutational changes that are required for tropism switch, but which usually come with costs to the virus because of fitness loss. Studies to monitor precisely the events surrounding coreceptor switch in the R5 SHIV infected RP macaques may provide insights into the driving forces for the virus to undergo a conformation change that exposes its receptor and coreceptor binding sites.

## Secondary lymphoid tissues are the sites of X4 virus evolution and emergence

Frequent samplings and tissue data are very limiting for HIV-1 infection of humans, highlighting the usefulness of the R5 SHIV monkey model in providing a detailed picture of the dynamics and anatomic sites of viral tropism change. In this regard, frequent and extensive samplings of lymphoid and nonlymphoid organs in R5 SHIV_SF162P3N_ infected RP macaques revealed different tempo and tissue localization of the emerging dual-tropic and X4 variants [89,113]. X4 viruses are poorly represented in the gut but are detectable in secondary lymphoid tissues such as the axillary, Iliac, and inguinal lymph nodes at the time of switch. This contrasts with dual-tropic viruses, which were easier to detect and had a much wider distribution, establishing infection in peripheral as well as mucosal lymphoid tissues. The greater representation and presence of the dual-tropic viruses in multiple tissue sites compared to the X4 virus indicated dominance and generalized dissemination of the former virus, and suggested that the dual-tropic switch event took place earlier than the X4 switch. Moreover, analysis of tissue samples collected at a time point that happened to capture a localized switch event in one infected RP macaque showed emergence of X4 viruses first in the inguinal lymph node [89]. Interestingly, in a study that examined envelope evolution in DKO-hu-HSC mice infected with the CCR5-tropic isolate HIV-1JRCSF, variants that could use CXCR4 in addition to CCR5 emerged in one mouse, and V3 sequences indicative of CXCR4-use were compartmentalized in the mesenteric lymph node [127]. As indicated above, CXCR4+CD4+ naïve T cells that are preferential targets for X4 viruses in acute infection are enriched in peripheral blood and secondary lymphoid tissues. Compartmentalization of these preferred targets for X4 viruses in peripheral lymph nodes, therefore, contribute to the regional evolution and selection of X4 viruses at these sites. Evolution and localization of X4 viruses that are neutralization sensitive in secondary lymphoid tissues which are not frequently sampled, together with the fact that immune selection against HIV appears to continue until late in infection [118,128], could explain the observation of X4 emergence only in a subset of patients progressing to AIDS.

## Concluding remarks

SIV and SHIV infection of RMs provide experimentally attractive models to study the impact of coreceptor usage on viral replication, CD4 T cell depletion and disease. Recent development of a simian model of coreceptor switching, based on the infection of rhesus macaques with pathogenic R5 SHIV isolates, further broadens the use of NHPs to study coreceptor switch following intravenous and mucosal infection. The conditions, phenotypic characteristics as well as envelope V3 sequences required for coreceptor switch in R5 SHIV infected macaques overlap with those reported in HIV-1 infected individuals, supporting the use of this model to study the mechanistic basis and selective forces for HIV-1 coreceptor switching in vivo. While this phenomenon has so far been documented only in a small number of R5 SHIV infected RPs who fail to mount or sustain virus-specific antibody response, studies of these animals are still important, for they allow examination of the process of a generalized switch uncomplicated by the selection pressure of antiviral immune responses. It remains to be determined how broadly findings in the SHIV-rhesus model of coreceptor switch relate to HIV infection of humans. Moreover, although humoral immune pressure may not be a main factor in driving coreceptor switch in the RP macaques, its presence could nevertheless shape or dictate the pathway of R5-to-X4 evolution. Variations in host genetic factors such as MHC, APOBEC and TRIM5 family of proteins [129-131] could also play roles in restricting the mutational pathways to coreceptor switch in vivo. Thus, the model could be improved by examining the process of coreceptor switching in genetically-defined R5 SHIV infected rhesus monkeys that have developed a neutralizing antibody response, to discern the impact of innate and adaptive immune selection forces on the evolutionary pathways available for tropism switch.

## Competing interests

The authors declared that no competing interests exist.
